# The New and Old Europe: East-West Split in Pharmaceutical Spending

**DOI:** 10.3389/fphar.2016.00018

**Published:** 2016-02-09

**Authors:** Mihajlo Jakovljevic, Marija Lazarevic, Olivera Milovanovic, Tatjana Kanjevac

**Affiliations:** ^1^Health Economics and Pharmacoeconomics, The Faculty of Medical Sciences, University of KragujevacKragujevac, Serbia; ^2^Department of Pharmacy, Faculty of Medical Sciences, University of KragujevacKragujevac, Serbia; ^3^Department for Preventive and Pediatric Dentistry, Faculty of Medicine, University of KragujevacKragujevac, Serbia

**Keywords:** Eastern Europe, Western Europe, centrally-planned economy, free market economy, pharmaceuticals, expenditure, spending, transition, I11 Analysis of Health Care Markets, I18 Government Policy Regulation Public Health, P2 Socialist Systems and Transitional Economies, P1 Capitalist Systems, P51 Comparative Analysis of Economic Systems, F6 Economic Impacts of Globalization

## Abstract

**HIGHLIGHTS**

- Since the geopolitical developments of 1989, former centrally planned economies of Eastern Europe followed distinctively different pathways in national pharmaceutical expenditure evolution as compared to their free market Western European counterparts.
- Long term spending on pharmaceuticals expressed as percentage of total health expenditure was falling in free market economies as of 1989. Back in early 1990s it was at higher levels in transitional Eastern European countries and actually continued to grow further.
- Public financing share of total pharmaceutical expenditure was steadily falling in most Central and Eastern European countries over the recent few decades. Opposed scenario were EU-15 countries which successfully increased their public funding of prescription medicines for the sake of their citizens.
- Pace of annual increase in per capita spending on medicines in PPP terms, was at least 20% faster in Eastern Europe compared to their Western counterparts. During the same years, CEE region was expanding their pharmaceuticals share of health spending in eight fold faster annual rate compared to the EU 15.
- Private and out-of-pocket expenditure became dominant in former socialist countries. Affordability issues coupled with growing income inequality in transitional economies will present a serious challenge to equitable provision and sustainable financing of pharmaceuticals in the long run.

## Introduction

Historical decades following WWII were marked with rapid industrialization and build-up of welfare states in most free-market economies. This trend was closely associated with health expenditures rising across 4% GDP threshold which remained quite stable throughout entire XIX and first half of XX century (Getzen, 1990). After this phenomenon was described in the US it became common elsewhere but most prominent in Western Europe, Japan and British Commonwealth countries. Over the next half a century health expenditure doubled or even tripled in many of the richest OECD societies. Challenges to sustainability of health care funding gradually were more obvious and concerning for policy makers. Unlike capital investments in buildings, equipment, medical staff salaries etc. prescription and dispensing of pharmaceuticals soon was understood to be more manageable part of these costs (Carone et al., 2012).

European geopolitical destiny since the end of Cold War Era back in 1989 opened up many issues ultimately affecting costs of medical care provision. Large number of previously state controlled socialist economies have undergone profound health reforms adopting free-market model (Jakovljevic, 2013). Central and Eastern European Post-Semashko Soviet style health systems were characterized with higher number of (more) hospital beds, physician, and nursing staff densities compared to Western Europe (Semashko, 1934; Torosyan et al., 2008). Nevertheless, average length of hospital stay was much lengthier and these nations had curative, hospital based systems instead of preventive ones, driven by family medicine practices common in the West (Healy and McKee, 2002). The latter turned out to be far more effective in terms of resource use and health outcomes gained (Kornai and Eggleston, 2001).

Evidence based medicine and cost-effective resource allocation slowly became more common in Eastern European policy makers mindset (Jakovljevic et al., 2011). These changes were closely to the rapid growth of most CEE pharmaceutical markets since the middle 1990s and early 2000s (Jakovljevic et al., 2015a). Although drug acquisition costs clearly grew up in the old EU-15 pre-2004 members as well, this appears to have happened at the far slower pace (Nuijten et al., 2001). Basically similar upward trends in value based turnover and budget impact of medicines in East and West of European region were hiding distinctively different patterns. We decided to observe WHO issued European Health for All database (HFA-DB) in order to test this assumption.

## Data report methods

### Public data sources used

WHO issued European Health for All database (HFA-DB) is a public registry with large number of data on demographics, health care resources and outcomes and medical service consumption data on all countries of the European Region (WHO, HFA-DB, 2015). It consists of regularly updated reports issued by WHO/European Office, the statistical office of the European Union (EUROSTAT), United Nations system, the Organization for Economic Cooperation and Development and data reported by the national authorities. Readers are free to access and reuse these publicly available data at the link provided beneath.

### Data

Pharmaceutical spending is commonly defined as expenditures on prescription medicines and over-the-counter products without hospital consumption of pharmaceuticals (OECD Pharmaceutical Spending, 2013). Medical consumables are included in such data in many countries (approximately 5% of reported value) and pharmacists' salaries if these are accounted separately from the price of medicines. Ultimately calculated total pharmaceutical expenditure assumes wholesale and retail margins and value-added tax.

Selected pharmaceutical spending indicators in this study were: Pharmaceutical expenditure expressed as percentage of total health expenditure, public pharmaceutical expenditure expressed as percentage of total pharmaceutical expenditure and pharmaceutical expenditure per capita expressed in purchase power parity terms (international $). Time horizon observed was spreading from the earliest available evidence listed in HFA-DB back in 1970 to the last official updates in 2012.Targeted countries where were all 53 countries of the European Region divided in two groups based on their economic and health care historical legacies: free-market economies prior to 1989 (a total of 25dominantly Western European countries) and centrally planned socialist economies prior to 1989 (a total of 28dominantly Eastern European countries; Berend, 2006). Total of five nations among free-market economies and ten among former centrally planned economies were observed for missing relevant data and thus were excluded from observation.

## Results

Pharmaceutical expenditure (PE) percentage of total health expenditure used to be higher in centrally planned economies. Mean of historical bottom values was 20% growing toward 21.7% in recent years. This meant total +3.0% net increase per country over 14.7 years long time horizon on average. Mean annual growth was calculated to be +0.08%. Unlike these, we notice exactly the opposed trend in free market economies. Their arithmetic mean of historical baseline values was 16.2%. It felt toward 15.1% in contemporary period. Net change here was negative: average −0.7% decrease over 33.5 years long time horizon on average. Length of observation here was longer because OECD economies pioneered reporting these data to WHO during the Cold War Era. Their mean annual growth was eight times slower, approximately +0.01%.

**Figure 1:**
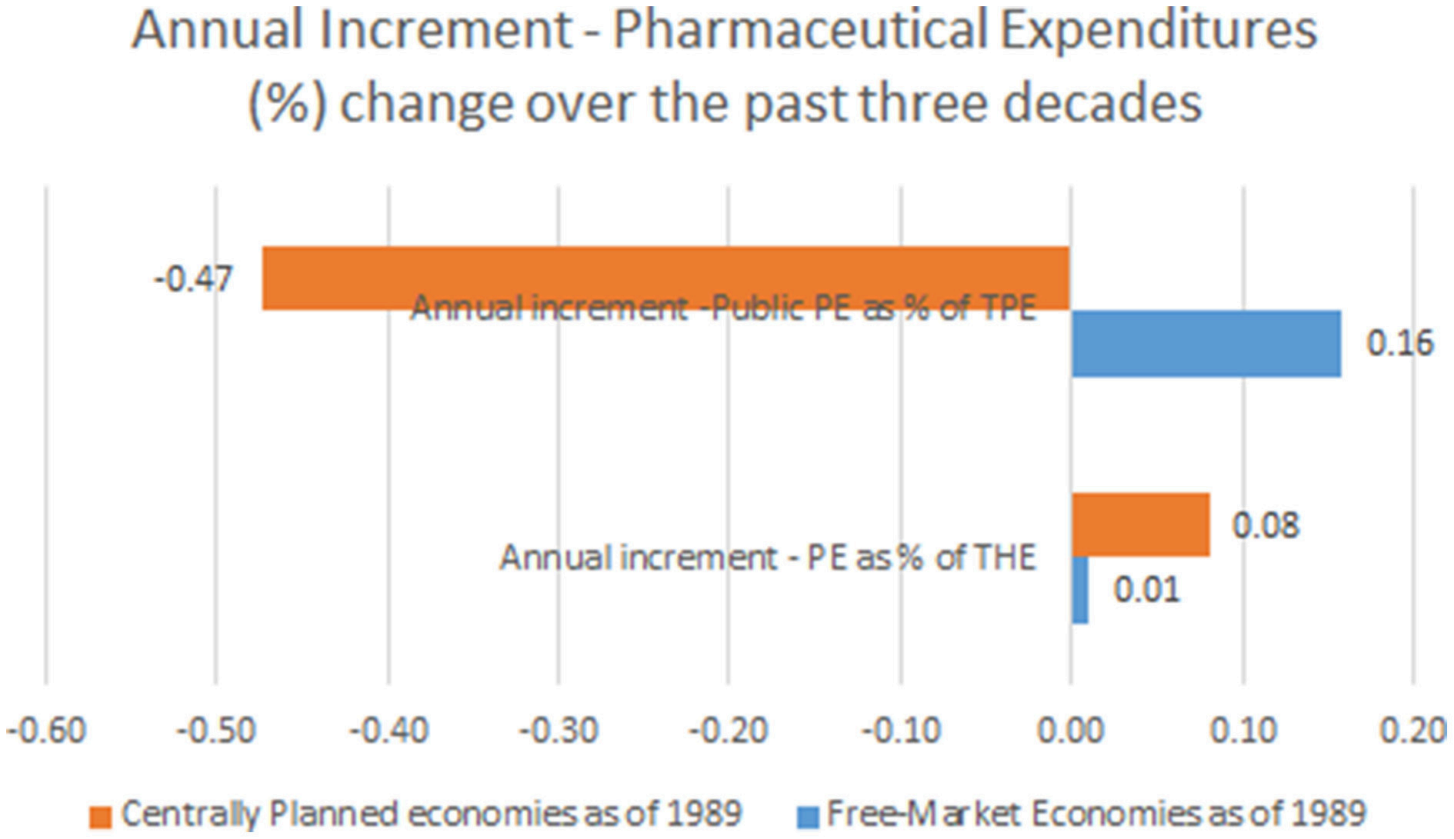
**Annual Increment—Pharmaceutical Expenditures (%) change (observation time span up to 22 years among former centrally planned and up to 42 years in free market economies)**.

Observing the landscape of public pharmaceutical expenditure percentage point share of total health expenditure we come to entirely different mirror-like reflection. Centrally planned economies tended to have lower public participation in drug acquisition and dispensing costs (arithmetic mean of earliest reported values of 43.3%) which was historically further falling toward 36.5% on average in recent years. This meant a total contraction of public spending on drugs of −6.8% on average. This huge change happened over the course of 10.6 years (mean time span between first and last reported values in Eastern European states).This accounts for mean annual −0.47% contraction of public spending on drugs. Free market nations recorded much higher average public share of 57.5% in earliest historical records back in 1970s. It has grown further up to 62.9% in recent years with a total net change of +5.4% over 32.3 years (mean time span between first and last reported values in Western, Southern and Northern European states). Annual net change was positive amounting to +0.16%. This huge disparity presents the single most important finding of this data report (see Figure 1).

Pharmaceutical expenditure per capita expressed in purchase power parity (PPP) terms (international $) allows for international comparability and these values introduce another perspective. Back in early 1970s free market economies were spending on medicines modest average amount of $68 per capita which grew tremendously reaching mean value of $488 in recent years. This meant net gain of $420 on average with annual increment of +$12.30 over 34.1 years (Table 1). Among most of the former centrally planned economies such data were lacking almost until the late 1990s. Therefore, we have come to a slightly distorted picture of mean $190 per capita among the earliest reported values rising up to $427 in recent years. Total increment of some $237 was approximately twice lower compared to the West. Keeping in mind that this change refers to the average time span of 13.8 years between the earliest and last reported values, we come to the annual increment of +$16.89 per capita (Table 2).

**Table 1:** **Pharmaceutical expenditure indicators according to WHO issued HFA-DBdata for free-market economies in the European Region prior to 1989 (5 out of 25 countries lacking some relevant data from the official records)**.

	First available PE^*^ as % of THE^**^	**Last available PE as % of THE**	**Total change PE as % of THE**	**Time span (years)**	**Annual increment**	First available public PE as % of TPE^***^	**Last available Public PE as % of TPE**	**Total change public PE as % of TPE**	**Time span (years)**	**Annual increment**	**First available PE as PPP$ per capita**	**Last available PE as PPP$ per capita**	**Total change PE as PPP$ per capita**	**Time span (years)**	**Annual increment**
Andorra	N/A	N/A	N/A	N/A	N/A	N/A	N/A	N/A	N/A	N/A	N/A	N/A	N/A	N/A	N/A
Austria	9.6^1990^	11.7^2011^	+2.1	21	0.10	55.1^1990^	67.6^2011^	+12.5	21	0.60	$156.5^1990^	$533.1^2011^	+$376.6	21	$17.93
Belgium	28.1^1970^	15.5^2011^	−12.6	41	−0.31	58.5^1970^	64.3^2011^	+5.8	41	0.14	$41.9^1970^	$630.9^2011^	+$589.0	41	$14.37
Cyprus	N/A	N/A	N/A	N/A	N/A	N/A	N/A	N/A	N/A	N/A	N/A	N/A	N/A	N/A	N/A
Denmark	N/A	6.8^2011^	N/A	N/A	N/A	49.9^1980^	49.2^2011^	−0.7	31	−0.02	$53.8^1980^	$300.4^2011^	+$246.6	31	$7.95
Finland	12.6^1970^	13.2^2011^	+0.6	41	0.01	33.7^1970^	55.9^2011^	+22.2	41	0.54	$22.8^1970^	$446.2^2011^	+$423.4	41	$10.33
France	23.8^1970^	15.6^2011^	−8.2	41	−0.20	67.3^1970^	68^2011^	+0.7	41	0.02	$46.1^1970^	$641.1^2011^	+$595.0	41	$14.51
Germany	16.2^1970^	14.1^2011^	−2.1	41	−0.05	63.4^1970^	75.6^2011^	+12.2	41	0.30	$43.3^1970^	$632.6^2011^	+$589.3	41	$14.37
Greece	25.5^1970^	28.5^2011^	+3.0	41	0.07	60^1970^	73.7^2011^	+13.7	41	0.33	$40.8^1970^	$673.4^2011^	+$632.6	41	$15.43
Iceland	17.1^1970^	15.4^2011^	−1.7	41	−0.04	43.2^1970^	42.1^2011^	−1.1	41	−0.03	$29.9^1970^	$508.3^2011^	+$478.4	41	$11.67
Ireland	13.7^1975^	17.5^2011^	+3.8	36	0.11	43.4^1975^	78^2011^	+34.6	36	0.96	$37.3^1975^	$647.7^2011^	+$610.4	36	$19.96
Israel	15.5^1995^	14.7^2011^	−0.8	16	−0.05	39.3^2006^	43.7^2010^	+4.4	4	1.10	N/A	N/A	N/A	N/A	N/A
Italy	21.8^1988^	15.7^2012^	−6.1	24	−0.25	61.5^1988^	45.5^2012^	−16.0	24	−0.67	$247.2^1988^	$482.2^2012^	+$235.0	24	$9.79
Luxembourg	19.7^1970^	9.1^2008^	−10.6	38	−0.28	83.5^1970^	84^2008^	+0.5	38	0.01	$229.1^1995^	$406^2008^	+$176.9	13	$13.61
Malta	23.69^2001^	27.98^2012^	+4.29	11	0.39	43.61^2001^	41.2^2012^	−2.41	11	−0.22	N/A	N/A	N/A	N/A	N/A
Monaco	N/A	N/A	N/A	N/A	N/A	N/A	N/A	N/A	N/A	N/A	N/A	N/A	N/A	N/A	N/A
Netherlands	10.3^1972^	9.4^2011^	−0.9	39	−0.02	60.1^1972^	78.4^2011^	+18.3	39	0.47	$32.3^1972^	$479.3^2011^	+$447.0	39	$11.46
Norway	7.8^1970^	6.6^2012^	−1.2	42	−0.03	35.8^1970^	54.5^2012^	+18.7	42	0.45	$11.2^1970^	$390.4^2012^	+$379.2	42	$9.03
Portugal	13.4^1970^	17.9^2011^	+4.5	41	0.11	68.7^1970^	55.1^2011^	−13.6	41	−0.33	$6.3^1970^	$469^2011^	+$462.7	41	$11.29
San Marino	N/A	N/A	N/A	N/A	N/A	N/A	N/A	N/A	N/A	N/A	N/A	N/A	N/A	N/A	N/A
Spain	21^1980^	17.4^2011^	−3.6	31	−0.12	64^1980^	71^2011^	+7.0	31	0.23	$76^1980^	$535.8^2011^	+$459.8	31	$14.83
Sweden	6.6^1970^	12.1^2011^	+5.5	41	0.13	62.9^1970^	58.3^2011^	−4.6	41	−0.11	$20.5^1970^	$474^2011^	+$453.5	41	$11.06
Switzerland	11.3^1985^	9.4^2011^	−1.9	26	−0.07	53.3^1995^	68.9^2011^	+15.6	16	0.98	$164.9^1985^	$530.7^2011^	+$365.8	26	$14.07
Turkey	10.7^1981^	26.6^2000^	+15.9	19	0.84	100^1981^	60.2^2000^	−39.80	19	−2.09	$9.1^1981^	$120.7^2000^	+$111.6	19	$5.87
United Kingdom	14.7^1970^	11.4^2008^	−3.3	38	−0.09	59.4^1970^	84.7^2008^	+25.3	38	0.67	$23.4^1970^	$374.6^2008^	+$351.2	38	$9.24
MEAN	16.2	15.1	−0.7	33.5	0.01	57.5	62.9	5.4	32.3	0.16	$68.0	$488.2	$420.2	34,1	$12.30
ST DEV	6.2	6.2	6.3	10.1	0.25	15.6	13.8	16.3	11.7	0.68	$74.0	$136.0	$150.5	9,2	$3.18
MIN	6.6	6.6	−12.6	11.0	−0.31	33.7	41.2	−39.8	4.0	−2.09	$6.3	$120.7	$111.6	13	$5.87
MAX	28.1	28.5	15.9	42.0	0.84	100.0	84.7	34.6	42.0	1.10	$247.2	$673.4	$632.6	42	$17.93
MEDIAN	15.1	14.7	−1.1	38.5	−0.04	59.4	64.3	5.8	38.0	0.23	$40.8	$482.2	$447.0	39	$11.67

* PE, Pharmaceutical expenditure.

** THE, Total health expenditure.

*** TPE, Total pharmaceutical expenditure.

**Table 2:** **Pharmaceutical expenditure indicators according to WHO issued HFA-DBdata for former centrally-planned socialist economies in the European Region prior to 1989 (10 out of 28 countries lacking some relevant data from the official records)**.

	First available PE^*^ as % of THE^**^	**Last available PE as % of THE**	**Total change PE as % of THE**	**Time PE as % (years)**	**Annual increment**	First available public PE as % of TPE^***^	**Last available Public PE as % of TPE**	**Total change public PE as % of TPE**	**Time span (years)**	**Annual increment**	**First available PE as PPP$ per capita**	**Last available PE as PPP$ per capita**	**Total change PE as PPP$ per capita**	**Time span (years)**	**Annual increment**
Armenia	N/A	N/A	N/A	N/A	N/A	N/A	N/A	N/A	N/A	N/A	N/A	N/A	N/A	N/A	N/A
Azerbaijan	N/A	26.2^2010^	N/A	N/A	N/A	32.9^2000^	20.4^2009^	−12.5	9	−1.39	N/A	N/A	N/A	N/A	N/A
Belarus	12.1^1990^	21^2012^	+8.9	22	0.40	20.4^1990^	44.8^2012^	+24.4	22	1.11	N/A	N/A	N/A	N/A	N/A
Bosnia and Herzegovina	8.9^1980^	27.4^2011^	+18.5	31	0.60	52.5^2009^	56.7^2011^	+4.2	2	2.10	N/A	N/A	N/A	N/A	N/A
Bulgaria	N/A	N/A	N/A	N/A	N/A	N/A	N/A	N/A	N/A	N/A	N/A	N/A	N/A	N/A	N/A
Croatia	N/A	N/A	N/A	N/A	N/A	N/A	N/A	N/A	N/A	N/A	N/A	N/A	N/A	N/A	N/A
Czech Republic	21^1990^	20^2011^	−1.0	21	−0.05	89^1990^	62.5^2011^	−26.5	21	−1.26	$115.2^1990^	$394.2^2011^	+$279.0	21	$13.29
Estonia	19.5^1999^	21.5^2012^	+2.0	13	0.15	40.8^1999^	49.2^2012^	+8.4	13	0.65	$100.4^1999^	$279.8^2011^	+$179.4	12	$14.95
Georgia	45.6^2000^	41.2^2011^	−4.4	11	−0.40	N/A	N/A	N/A	N/A	N/A	N/A	N/A	N/A	N/A	N/A
Hungary	27.6^1991^	33.4^2011^	+5.8	20	0.29	79.3^1991^	49^2011^	−30.3	20	−1.52	$158.6^1991^	$564^2011^	+$405.4	20	$20.27
Kazakhstan	6.8^1991^	2.8^2000^	−4.0	9	−0.44	8.3^1991^	1.6^1997^	−6.7	6	−1.12	N/A	N/A	N/A	N/A	N/A
Kyrgyzstan	10.9^1990^	11.2^2002^	+0.3	12	0.03	9.9^1992^	9.9^1992^	0	0	N/A	N/A	N/A	N/A	N/A	N/A
Latvia	21.9^2005^	24.2^2010^	+2.3	5	0.46	31.3^2005^	38.4^2010^	+7.1	5	1.42	N/A	N/A	N/A	N/A	N/A
Lithuania	32.9^2004^	24.92^2011^	−7.98	7	−1.14	34.96^2004^	34.22^2011^	−0.74	7	−0.11	N/A	N/A	N/A	N/A	N/A
Montenegro	N/A	N/A	N/A	N/A	N/A	N/A	N/A	N/A	N/A	N/A	N/A	N/A	N/A	N/A	N/A
Poland	28.4^2002^	22.5^2011^	−5.9	9	−0.66	38.4^2002^	39.4^2011^	+1.0	9	0.11	$207.9^2002^	$326.3^2011^	+$118.4	9	$13.16
Republic of Moldova	7.94^1991^	32.9^2012^	+24.96	21	1.19	9.1^1993^	6.4^2012^	−2.7	19	−0.14	N/A	N/A	N/A	N/A	N/A
Romania	N/A	20^1998^	N/A	N/A	N/A	N/A	N/A	N/A	N/A	N/A	N/A	N/A	N/A	N/A	N/A
Russian Federation	N/A	N/A	N/A	N/A	N/A	N/A	N/A	N/A	N/A	N/A	N/A	N/A	N/A	N/A	N/A
Serbia	11.83^1998^	31.3^2011^	19.47	13	1.50	50.4^2003^	44.7^2011^	−5.7	8	−0.71	N/A	N/A	N/A	N/A	N/A
Slovakia	34^1999^	27.4^2011^	−6.6	12	−0.55	76.2^1999^	69.4^2011^	−6.8	12	−0.57	$203.8^1999^	$525^2011^	+$321.2	12	$26.77
Slovenia	20.9^2002^	19.5^2011^	−1.4	9	−0.16	61.5^2002^	55.9^2011^	−5.6	9	−0.62	$355.3^2002^	$471.3^2011^	+$116.0	9	$12.89
Tajikistan	13^1991^	10.5^2003^	−2.5	12	−0.21	N/A	N/A	N/A	N/A	N/A	N/A	N/A	N/A	N/A	N/A
TFYR Macedonia	13.8^1980^	14.9^2004^	+1.1	24	0.05	N/A	N/A	N/A	N/A	N/A	N/A	N/A	N/A	N/A	N/A
Turkmenistan	N/A	N/A	N/A	N/A	N/A	N/A	N/A	N/A	N/A	N/A	N/A	N/A	N/A	N/A	N/A
Ukraine	N/A	4.2^2009^	N/A	N/A	N/A	95.3^1993^	34.9^2005^	−60.4	12	−5.03	N/A	N/A	N/A	N/A	N/A
Uzbekistan	N/A	11.6^2001^	N/A	N/A	N/A	6.4^1993^	3.4^1999^	−3.0	6	−0.50	N/A	N/A	N/A	N/A	N/A
MEAN	20.0	21.7	3.0	14.7	0.08	43.3	36.5	−6.8	10.6	−0.47	$190.2	$426.8	$236.6	13.8	$16.89
ST DEV	10.6	9.6	9.4	6.9	0.64	28.9	21.2	18.6	6.6	1.60	$92.1	$112.4	$117.7	5.3	$5.58
MIN	6.8	2.8	−8.0	5.0	−1.14	6.4	1.6	−60.4	0.0	−5.03	$100.4	$279.8	$116.0	9.0	$12.89
MAX	45.6	41.2	25.0	31.0	1.50	95.3	69.4	24.4	22.0	2.10	$355.3	$564.0	$405.4	21.0	$26.77
MEDIAN	20.2	22.0	0.7	12.5	0.04	38.4	39.4	−3.0	9.0	−0.53	$181.2	$432.8	$229.2	12.0	$14.12

* PE, Pharmaceutical expenditure.

** THE, Total health expenditure.

*** TPE, Total pharmaceutical expenditure.

## Discussion

Long term spending on pharmaceuticals expressed as percentage of total health expenditure was falling in mature economies (Mossialos and Oliver, 2005). Back in the late 1980s and early 1990s it was at higher levels in transitional Eastern European countries and actually continued to grow (Mrazek et al., 2004). Opposed to this trend, free market, predominantly Western, Southern and Northern European states continued to contract participation of medicines in their national medical spending pattern. This effectively meant that other medical technologies mostly related to hospital care, such as radiology diagnostics, advanced surgery, interventional radiology and radiation oncology, laboratory tests, rehabilitating and mental health related medical services and social support programs were participating more significantly to the structure of medical spending (Robinson, 1994; Ackroyd et al., 2006; Jakovljevic et al., 2013, 2014a, 2015b; Ranković et al., 2013).

Public financing share of total pharmaceutical expenditure was steadily falling in most Central and Eastern European countries (Gotseva, 2015). It was at far lower, approximately one-third level, compared to their Western counterparts back in Cold War era (Jakovljevic et al., 2015d). Today, such changes coupled with rising budget impact of drug acquisition costs point out to the strong growth of patient cost-sharing mechanisms throughout the CEE region (Iskrov and Stefanov, 2015; Tambor et al., 2015). Out-of-pocket expenses and risks of catastrophic illness-induced household expenditure add to the complexity of this challenge (Jakovljevic, 2014a,b).

Opposed scenario were EU-15 countries successfully struggling to maintain and increase their public funding of prescription medicines for the sake of their citizens. They achieved extension in population coverage with cost-effective drug reimbursement strategies (Rémuzat et al., 2015). Thus, these countries were reducing exposure of vulnerable social groups to the issues affecting access to medicines. Although success rates across EU-15 differ significantly, most countries have adopted responsible pharmaceutical polices. National authorities besides, proved mostly capable of withstanding diverse financial constraints. Some were temporary such as the global economic recession while others such as medical innovation led primarily by brand pharmaceutical industry posed difficulties in the long run (Higgins and Graham, 2009; Dagovic et al., 2015). Huge budget impact of novel medicines such as monoclonal antibodies remains particularly hot topic sparkling debate among policy makers (Jakovljevic, 2014b). Some of the solutions found to release such pressures were incentives for generic substitution of brand name medicines (Jakovljevic et al., 2014b). With more or less legislative obstacles generic share in local markets expanded significantly over time (Simoens and De Coster, 2006).

Per capita spending on pharmaceuticals expressed in purchased power parity terms, points out to the joint strong growth of overall costs of prescribed and dispensed medicines and OTC agents (Ess et al., 2003). With regards to the historical perspective there appears to be no distinct difference in spending patterns among the two regions. The obvious fact was lag in Eastern European drug acquisition costs back in the late 1980s (Rhodes et al., 1999). Rapid increase in pharmaceutical expenditure since the 1990s followed, being one of the recognized mile stones of transitional health care reforms (Krajewski-Siuda and Romaniuk, 2006). Notable transformation of local CEE markets is the expanded presence of brand name medicines and diversification of payment mechanisms (Petrusic and Jakovljevic, 2015). Informal payments and widening income gaps affecting affordability of medicines remain the key challenges across the region (Ensor, 2004; Jakovljevic et al., 2015c).

## Study limitations

Some countries did not report official data on pharmaceutical spending for either some indicators or years within the time span observed. These missing data refer to a total of 5 nations among free-market economies and 10 countries among former centrally planned economies. Most cases of partially or entirely missing data refer to the countries with relatively small population size in respective groups. Notable exceptions from this rule are Romania and Russian Federation. Russia was classified as a high income economy by the World Bank since August 2013 and as one of top performing emerging BRICS markets (Jakovljevic, 2016; The World Bank, 2015). Due to these facts we would like to limit our results and conclusions on the rest of Eastern European region as thus there is higher degree of homogeneity. Countries presented in the observed sample (20 of historical free market and 18 of centrally planned economies) are geographically scattered throughout the respective European regions. Therefore, we still regard observed sample of countries to be representative of broad long term trends in pharmaceutical spending in the European Region.

## Conclusion

The two observed broad groups of countries, former centrally planned and free market economies, share profoundly different historical legacies in medicines provision and financing mechanisms (Mossialos et al., 2004). This data report provides insight into the existence of two distinctively different pathways in spending on drugs over past several decades in the East and West of Europe. National policies sharing public reimbursement, insurance based mechanisms and out-of-pocket spending appear to be headed in two different directions (Mackenbach, 2006). Eastern European states struggle with affordability issues and unequal access to medicines mostly determined by household income groups. Single most concerning fact is that transitional economies contracted their public share of pharmaceutical expenditure for almost half of percentage over the long course of years. Traditional free market economies, primarily EU-15 states mostly achieved better protection for their poor and vulnerable patient groups including those suffering from rare diseases and those requiring expensive treatment strategies (Iskrov et al., 2012). Nevertheless, we must point out the huge progress that was made in Eastern Europe providing access to the innovative medicines to the broad layers of population (Putrik et al., 2014). Pace of annual increase in per capita spending on medicines in PPP terms, was at least 20% faster in Eastern Europe compared to their Western counterparts. During the same years CEE region was expanding their pharmaceuticals share of health spending in eight fold faster annual rate compared to the EU 15. Current difficulties to withstand pressures arising from population aging and prosperity diseases remain primary challenge for sustainable funding of medicines provision in all of Europe (Ogura and Jakovljevic, 2014; Jakovljevic and Milovanovic, 2015; Jakovljevic and Laaser, 2015). Although differences remain we believe that at some point in future, these regions will converge increasing social welfare and affordability of medicines to the ordinary citizens (Deacon, 2000).

## Author contributions

MJ and TK developed research questions, designed the study and drafted most of the manuscript. ML and OM took part in data acquisition, mining and analysis and prepared the tables and figure. All four authors revised draft and contributed essentially to the final appearance of the manuscript.

### Conflict of interest statement

The authors declare that the research was conducted in the absence of any commercial or financial relationships that could be construed as a potential conflict of interest.
